# Crucial Role of Membrane Potential in Heat Stress-Induced Overproduction of Reactive Oxygen Species in Avian Skeletal Muscle Mitochondria

**DOI:** 10.1371/journal.pone.0064412

**Published:** 2013-05-09

**Authors:** Motoi Kikusato, Masaaki Toyomizu

**Affiliations:** Animal Nutrition, Life Sciences, Graduate School of Agricultural Science, Tohoku University, Sendai, Japan; Instituto de Investigación Hospital 12 de Octubre, Spain

## Abstract

Heat stress is an environmental factor that causes oxidative stress. We found previously that acute heat stress stimulates the production of reactive oxygen species (ROS) in the skeletal muscle mitochondria of birds, and that this was accompanied by an increase of the mitochondrial membrane potential (ΔΨ) due to increased substrate oxidation by the electron transport chain. We also showed that avian uncoupling protein (avUCP) expression is decreased by the heat exposure. The present study clarifies whether ΔΨ is a major determinant of the overproduction of ROS due to acute heat stress, and if the decrease in avUCP expression is responsible for the elevation in ΔΨ. Control (24°C) and acute heat-stressed (34°C for 12 h) birds exhibited increased succinate-driven mitochondrial ROS production as indicated by an elevation of ΔΨ, with this increase being significantly higher in the heat-stressed group compared with the control group. In glutamate/malate-energized mitochondria, no difference in the ROS production between the groups was observed, though the mitochondrial ΔΨ was significantly higher in the heat-stressed groups compared with the control group. Furthermore, mitochondria energized with either succinate/glutamate or succinate/malate showed increased ROS production and ΔΨ in the heat-stressed group compared with mitochondria from the control group. These results suggest that succinate oxidation could play an important role in the heat stress-induced overproduction of mitochondrial ROS in skeletal muscle. In agreement with the notion of a decrease in avUCP expression in response to heat stress, proton leak, which was likely mediated by UCP (that part which is GDP-inhibited and arachidonic acid-sensitive), was reduced in the heat-exposed group. We suggest that the acute heat stress-induced overproduction of mitochondrial ROS may depend on ΔΨ, which may in turn result not only from increased substrate oxidation but also from a decrease in the mitochondrial avUCP content.

## Introduction

Oxidative stress resulting from an imbalance between anti-oxidative capacity and reactive oxygen species (ROS) generation, is associated with many pathological processes, neurodegenerative diseases and ageing [1]. Given that ROS are produced to a large extent by mitochondria [2]–[4], the overproduction of mitochondrial ROS could be a major determinant of oxidative stress. A mechanistic understanding of the overproduction of mitochondrial ROS under abnormal conditions is therefore of significant importance. Heat stress is one of a range of environmental factors responsible for oxidative stress in birds [5], [6] and mammals [7], [8]. We have previously found that acute heat stress stimulates mitochondrial ROS production [9], causing oxidative damage to the skeletal muscle of birds [6].

There is considerable interest in the mechanism by which acute heat stress enhances mitochondrial ROS production in skeletal muscle. It is well accepted that complexes I and III of the mitochondrial electron transport chain are major sites of ROS production [10], [11]. Mitochondrial ROS production at complex I (energized by complex II-linked substrates) is highly sensitive to the mitochondrial membrane potential (ΔΨ), probably due to reverse electron flow from coenzyme Q to complex I [12]–[15]. Indeed, we previously reported that mitochondrial ROS production in heat-stressed birds was significantly increased when mitochondria were energized with succinate as a complex II-linked substrate [16], [17]; this was accompanied by an increase in mitochondrial ΔΨ [16], [17]. One could postulate that the heat stress-induced overproduction of ROS during succinate oxidation may be due to the increase of ΔΨ. However, there is no direct evidence regarding the dependence of ROS overproduction on ΔΨ in the skeletal muscle mitochondria of acute heat-stressed birds. Furthermore, we reported that mitochondrial ROS production with glutamate/malate as complex I-linked substrates was increased by heat exposure [9], [18], [19], but it remains unclear if the overproduction of ROS depends on the magnitude of ΔΨ.

Acute heat stress induces certain changes relevant to the elevation of ΔΨ. We previously found that substrate oxidation by the electron transport chain, which is a ΔΨ-producer, was not only significantly increased, presumably resulting in an increase of ΔΨ in the muscle mitochondria of acute heat-stressed birds [17], but also that the heat stress significantly decreased the mitochondrial content of avUCP [18]. UCPs are mitochondrial inner membrane proteins that allow the passive transport of protons from the intermembrane space into the matrix. This transport activity leads to the uncoupling of mitochondrial oxidative phosphorylation [20]. It has been proposed that mild uncoupling caused by UCPs can decrease mitochondrial ROS production by lowering the proton motive force (Δp) and the local oxygen concentration [12], [21]. Indeed, transgenic mice lacking UCP3 exhibited an increased ΔΨ and ROS production compared to wild-type mice [22]. Therefore, it can be postulated that the decrease in avUCP protein content in heat-stressed birds may be also involved in the elevation of ΔΨ and concomitant overproduction of ROS, possibly via the downregulation of proton leak. However, this possibility has not yet been determined experimentally.

In this study, we determine *i*) whether ΔΨ is a major determinant of the overproduction of mitochondrial ROS induced by acute heat stress, and *ii*) if a decrease in avUCP content is accompanied by a reduction of proton leak in the skeletal muscle mitochondria of birds exposed to acute heat stress. We show that ΔΨ may play a crucial role in the overproduction of mitochondrial ROS under heat stress conditions.

## Materials and Methods

### Ethics statement

The Animal Care and Use Committee of the Graduate School of Agricultural Science, Tohoku University, approved all procedures, and every effort was made to minimize pain or discomfort to the animals.

### Animals and experimental design

One-day-old male chicks (Cobb strain, *Gallus gallus domesticus*) were obtained from a commercial hatchery (Economic Federation of Agricultural Cooperatives, Iwate, Japan). They were housed in electrically-heated batteries under continuous light, and provided with *ad libitum* access to water and a commercial starter meat-type chick diet (crude protein, 23%; metabolizable energy content, 3050 kcal/kg). After 14 days, the birds were randomly divided into two groups (n = 12 birds per group). After a further 7-day adaptation period, one of the two groups was exposed to 34°C for 12 h, while the other group was maintained at 24°C (humidity 55±5%). Birds were provided with *ad libitum* access to water and diet during the heat treatment. Thereafter, eight birds in each group were selected randomly and killed by decapitation. This method of killing was used in preference to overdose by general anesthetics, which are known to uncouple oxidative phosphorylation [23]. *Pectoralis superficialis* muscles were rapidly excised, and a sample of each muscle was placed in ice-cold isolation medium comprised of 100 mM KCl, 50 mM Tris/HCl (pH 7.4), and 2 mM EGTA for mitochondrial isolation (see below). Muscles were frozen, powdered in liquid nitrogen, and stored at –80°C until required for TBARS and protein carbonyl assays.

### Isolation of skeletal muscle mitochondria

Muscle mitochondria were isolated by homogenization, protein digestion and differential centrifugation at 4°C, as described previously [16], [24]. Muscle was trimmed of fat and connective tissue, blotted dry, weighed, and placed in isolation medium on ice. Tissue was shredded and minced with sharp scissors, rinsed with isolation medium three times, stirred for 5 min in protein digestion medium (100 mM KCl, 50 mM Tris/HCl (pH 7.4), 2 mM EGTA, 1 mM ATP, 5 mM MgCl_2_, 0.5% (*w*/*v*) bovine serum albumin (BSA), and 11.8 units of protease (subtilisin type VIII, Sigma) per gram of tissue). Muscles were gently homogenized with a Polytron PT-3100 (Kinematica) 3 times for 6 s at speed setting 7 (7,000 rpm.) The homogenate was stirred for 6 min before being mixed with the equivalent medium without protease to stop protease activity. The homogenate was rehomogenized in a Potter-Elvehjem homogenizer (six passages) and centrifuged at 500×*g* for 10 min. The supernatant was filtered through muslin and centrifuged at 10,400×*g* for 10 min. Mitochondrial pellets were resuspended in isolation medium and centrifuged at 10,400×*g* for 10 min, followed by 3,800×*g* for 10 min, and then resuspended in isolation medium. The mitochondrial protein concentration was determined using the bicinchoninic acid (BCA) assay, with BSA as the standard [25]. One sample of mitochondria was isolated from the pooled muscles of two birds, and four samples of mitochondria were isolated for each group. All mitochondria were freshly prepared on the day of the experiment, and respiratory control ratio (RCR: ratio of O_2_ consumption rate in state 3 to state 4) of each mitochondrial sample was routinely checked to examine mitochondrial quality. RCR values were 3.9±0.4 and 4.7±0.3 for control and heat-stressed groups, respectively, indicating that the isolated mitochondria were of good quality for evaluating their function. The mitochondrial samples were stored at −80°C until required for the immunodetection of avUCP protein content.

### Measurements of mitochondrial O_2_ consumption rate and membrane potential (ΔΨ)

Mitochondrial O_2_ consumption rate and ΔΨ were measured simultaneously using an oxygen-sensitive electrode and a potential-dependent probe (triphenylmethyl phosphonium cation (TPMP^+^)), respectively [24]. Briefly, O_2_ consumption was measured using a Clark-type oxygen electrode (Rank Brothers, Cambridge, UK) maintained at 38°C and calibrated with air-saturated assay medium, which were assumed to contain 402 nmol of atomic oxygen per milliliter [26]. Mitochondria were energized with 10 mM glutamate, 2.5 mM malate, or 4 mM succinate alone or in combination to initiate respiration. The TPMP^+^ electrode was calibrated with sequential additions of 0.5 up to 2.0 μM TPMP^+^. The electrode linearity was routinely checked by following uncoupled respiration in the presence of 1.0 μM carbonyl cyanide *p*-trifluoromethoxyphenyl hydrazone (FCCP) from 100% to 0% air saturation. This uncoupler dissipated ΔΨ and released TPMP back into the medium, allowing for the correction of any small electrode drift. ΔΨ values were calculated from the distribution of TPMP^+^ across the mitochondrial inner membrane, using a binding correction factor of 0.45 mg protein/μl [27].

### Kinetic response of substrate oxidation and basal proton leak to ΔΨ

Substrate oxidation by the mitochondrial electron transport chain and basal proton leak were measured as described previously [17]. Briefly, mitochondria (0.35 mg protein/ml) were incubated at 38°C in assay medium A (115 mM KCl, 10 mM KH_2_PO_4_, 3 mM HEPES (pH 7.2), 1 mM EGTA, 2 mM MgCl_2_ and 0.3% (*w*/*v*) defatted BSA) containing 0.1 μM nigericin (to collapse ΔpH) and 1 μg/ml oligomycin (to inhibit residual ATP synthesis), to which 4 mM succinate was added to initiate respiration. To obtain the kinetic response of the substrate oxidation to ΔΨ, state 4-respiration (non-phosphorylation state) was titrated with sequential additions of FCCP (up to 0.6 μM). In addition, to obtain the kinetic response of the basal proton leak to ΔΨ, state 4-respiration was also titrated with malonate (up to 3.2 mM). For the measurement of these kinetic responses, rotenone (which prevents reverse electron flow transport during FADH_2_ oxidation) was not added to the assay medium.

### Kinetic response of free fatty acid-induced proton leak to ΔΨ

To precisely evaluate the effect on proton leak of a decrease in avUCP protein content due to acute heat stress, arachidonic acid and GDP were employed. Briefly, mitochondria were incubated at 38°C in assay medium B (80 mM KCl, 50 mM Hepes (pH 7.2), 1 mM EGTA, 5 mM K_2_HPO_4_, 5 mM MgCl_2_, 0.1% (*w*/*v*) defatted BSA) containing 0.1 μM nigericin and 1 μg/ml oligomycin and energized with 4 mM succinate. Arachidonic acid (final concentration of 36 μM) was then added to the mitochondrial suspension to induce the uncoupling activity. Thereafter, mitochondria were titrated with increasing amounts of malonate (up to 3.2 mM) in the same manner as that used to measure the kinetics of basal proton leak. Following this, GDP (500 μM), which is a strong inhibitor of UCP, was added to inhibit avUCP-mediated uncoupling activity, and the GDP-sensitive portion of the arachidonic acid-induced proton leak was estimated. In this study, rotenone was eliminated from the assay medium in order to induce endogenous superoxide production at complex I, because UCP requires either free fatty acids or superoxide to exert the uncoupling effect [28], [29].

### Determination of mitochondrial ROS production and free radical leak

Mitochondrial hydrogen peroxide (H_2_O_2_) generation rates were determined fluorometrically by measurement of the oxidation of 10-acetyl-3,7-dihydroxyphenoxazine (amplex red, Invitrogen) coupled to the enzymatic reduction by horseradish peroxidase (HRP) as previously described [17]. This was performed under similar conditions to those used for the O_2_ consumption measurements. Briefly, H_2_O_2_ released from mitochondria was detected by 50 μM amplex red in the presence of 6 U/ml HRP and 30 U/ml superoxide dismutase (SOD). The rate of H_2_O_2_ production was spectrofluorimetrically determined by the change in fluorescence at excitation and emission wavelengths of 544 and 590 nm, respectively. In addition, the present study addressed to determine the response of ROS production to both ΔΨ and the O_2_ consumption rate for the substrate oxidation and basal proton leak kinetics, mitochondria were titrated with FCCP (up to 0.6 μM) and malonate (up to 3.2 mM) during fluorescence measurement of the mitochondrial H_2_O_2_ production, respectively. The ΔΨ value, O_2_ consumption rate and H_2_O_2_ production rate were plotted on X-, Y- and Z-axes, respectively, and presented in a three-dimensional diagram.

Mitochondrial H_2_O_2_ production and O_2_ consumption rates were measured in parallel under the same experimental conditions, which allowed the mitochondrial free radical leak (FRL) to be calculated. FRL (expressed as a percentage) was calculated as described previously [30]; it provides a measure of the number of electrons that produce superoxide (and subsequently H_2_O_2_) compared with the total number of electrons that pass through the respiratory chain. Two electrons are required to reduce 1 mol of O_2_ to H_2_O_2_, whereas four electrons are transferred in the reduction of 1 mol of O_2_ to water; in this way, the percent free radical leak is calculated as the rate of H_2_O_2_ production divided by twice the rate of O_2_ consumption, and the result multiplied by 100.

### Quantification of avUCP protein using Western blot analysis

Western blot analysis for mitochondrial avUCP was carried out as described previously [31].

### Determination of oxidative stress

Lipid peroxidation was measured as a marker of oxidative stress. Muscle homogenates were assayed for 2-thiobarbituric acid reactive substances (TBARS) as a conventional index for lipid peroxidation. The TBARS assay was conducted as described previously [6]. In addition, protein carbonyl content, which is also one of major indexes of oxidative damage, in skeletal muscle was spectrophotometrically determined by derivatization with 2,4-dinitrophenylhydrazone (DNPH) as described previously [32] with minor modifications.

### Statistical Analysis

All data are presented as means ± S.E. for 4 replicates (for mitochondrial function and protein) or 8 replicates (TBARS and protein carbonyl assays) of individual measurements. Statistical significance between control and heat-stressed groups was assessed using the Mann-Whitney *U* test. Differences were considered significant for values of *P*<0.05.

## Results

### Oxidative damage in skeletal muscle

**Figure 1:**
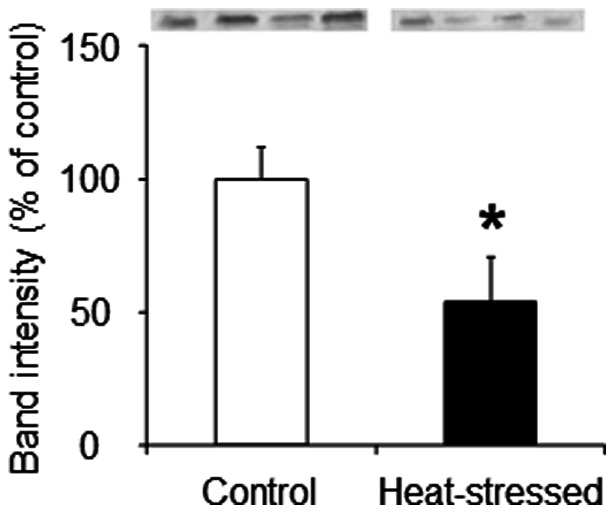
Lipid peroxidation (A) and protein carbonyl (B) in the *pectoralis superficialis* muscle. Lipid peroxidation was colorimetrically determined using a conventional TBARS assay. To determine protein carbonyl content by derivatization with DNPH, an additional experiment was similarly conducted. All measurements were carried out as described in the Materials and Methods. Values are means ± SE of data from eight individual tissue samples. *P<0.05 compared to control group.

In agreement with a previous study [6], the TBARS content of muscles from heat-stressed birds was significantly increased compared with that for the control group (Fig. 1A). Furthermore, the protein carbonyl content was also significantly increased by the heat exposure (Fig. 1B). These increases are highly likely to be due to the overproduction of mitochondrial ROS in the skeletal muscle of the heat-stressed birds. We therefore focused our attention on the mitochondrial bioenergetic function that results in the overproduction of ROS in the heat-stressed birds.

### Kinetic response of substrate oxidation and basal proton leak to ΔΨ, and ROS production

**Figure 2:**
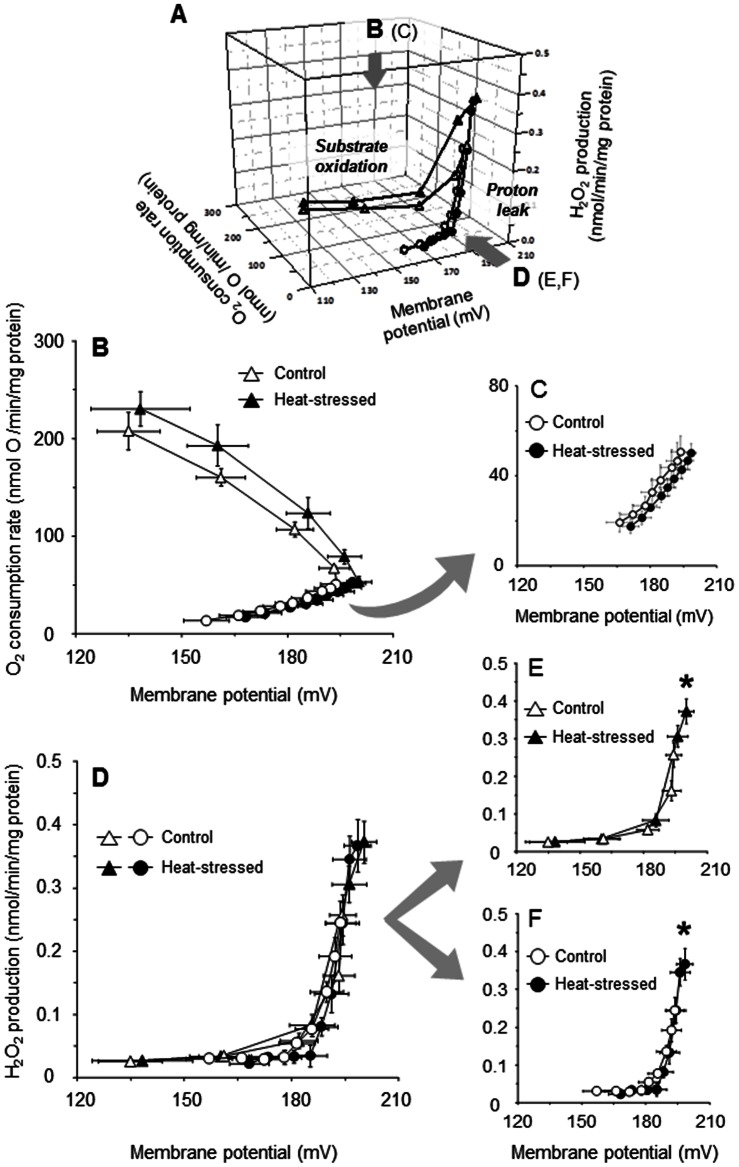
Three-dimensional diagram showing ROS (H_2_O_2_) production, ΔΨ and O_2_ consumption rate for substrate oxidation (triangles) and basal proton leak (circles) in skeletal muscle mitochondria of control (open symbols) and heat-stressed (closed symbols) groups. Kinetic responses of substrate oxidation (B) and basal proton leak (B and C, enlarged) as a function of ΔΨ. (D) ROS production as a function of ΔΨ for substrate oxidation (E) and basal proton leak (F). Mitochondria (0.35 mg protein/ml) were incubated at 38°C in assay medium A containing 0.1 μM nigericin and 1 μg/ml oligomycin, to which 4 mM succinate was added to initiate respiration. To obtain the kinetics of substrate oxidation and basal proton leak, mitochondrial state 4 respiration was titrated with FCCP (up to 0.6 μM) and malonate (up to 3.2 mM), respectively. Mitochondrial ROS production was determined using 50 μM amplex red in the presence of 6 U/ml HRP and 30 U/ml SOD. All measurements were carried out as described in the Materials and Methods. Values are means ± S.E. of data from four individual measurements in each group. *P<0.05 compared with the controls in the ROS production.

Figure 2 shows the kinetic responses of substrate oxidation and basal proton leak to ΔΨ, and H_2_O_2_ production in skeletal muscle mitochondria isolated from control and heat-stressed birds. The kinetics and H_2_O_2_ production in mitochondria were measured using the same assay medium as that used to measure O_2_ consumption, however rotenone was omitted in order to enable precise evaluation of the response of ROS production to ΔΨ. As shown in Fig. 2A, it can be seen that mitochondrial H_2_O_2_ production for the substrate oxidation and basal proton leak kinetics was highly sensitive to ΔΨ in both the control and heat-stressed groups. It can also be appreciated that the H_2_O_2_ production rate at the furthest point (state 4) to the right in each kinetic curve was appreciably higher for the heat-stressed group than for the control group, which suggests that mitochondria isolated from the heat-stressed group may have an increased ΔΨ in state 4 compared to that of controls (Fig. 2A). In fact, as shown in Fig. 2B, ΔΨ in state 4 for the substrate oxidation and basal proton leak kinetics showed a definite trend towards higher values in the heat-stressed group than in the control group. This finding suggests that the increase of ΔΨ in state 4 may be involved in the overproduction of mitochondrial ROS in the skeletal muscle of heat-stressed birds.

Figure 2B shows that the O_2_ consumption rate due to the substrate oxidation was tended to be enhanced at any given ΔΨ for the heat exposure condition. In contrast, the O_2_ consumption rate due to the basal proton leak was slightly decreased in the heat-stressed group compared with the control group (Fig. 2B and 2C, enlarged). Considering the suggestion that mitochondrial state 4 respiration is shared with substrate oxidation as a ΔΨ-producer and/or proton leak as a ΔΨ-consumer [27], the heat stress-induced increase of ΔΨ in state 4 may result from increased substrate oxidation and decreased basal proton leak (Fig. 2B). In agreement with previous studies [16], [17], an increase in the substrate oxidation and ΔΨ in state 4 for the heat-stressed group was also observed when mitochondria were incubated in the presence of rotenone, while no difference in basal proton leak between the control and heat-stressed groups was observed for this condition (data not shown).

### Dependence of mitochondrial ROS production on ΔΨ

The relationships between mitochondrial H_2_O_2_ production and ΔΨ for substrate oxidation and basal proton leak kinetics in control and heat-stressed birds are shown in Fig. 2D–F. Figure 2D shows that the H_2_O_2_ production was not only positive but also exponentially sensitive to ΔΨ in both the control and heat-stressed groups, even though only small differences in H_2_O_2_ production are evident between the substrate oxidation and basal proton leak kinetics. For both experimental groups, the highest H_2_O_2_ production rate was observed at the furthest point (state 4) to the right in the curves for each kinetic response (Fig. 2E, F). Moreover, the H_2_O_2_ production rate at state 4 was significantly higher for the heat-stressed group than for the control group, probably because of the larger ΔΨ generated in the heat-stressed group. In addition, there were only small differences in H_2_O_2_ production for the substrate oxidation and basal proton leak kinetics in each group at any given ΔΨ (Fig. 2D). It thus seems reasonable to assume that the overproduction of skeletal muscle mitochondrial ROS may result from an increase in ΔΨ in birds exposed to acute heat stress.

**Figure 3:**
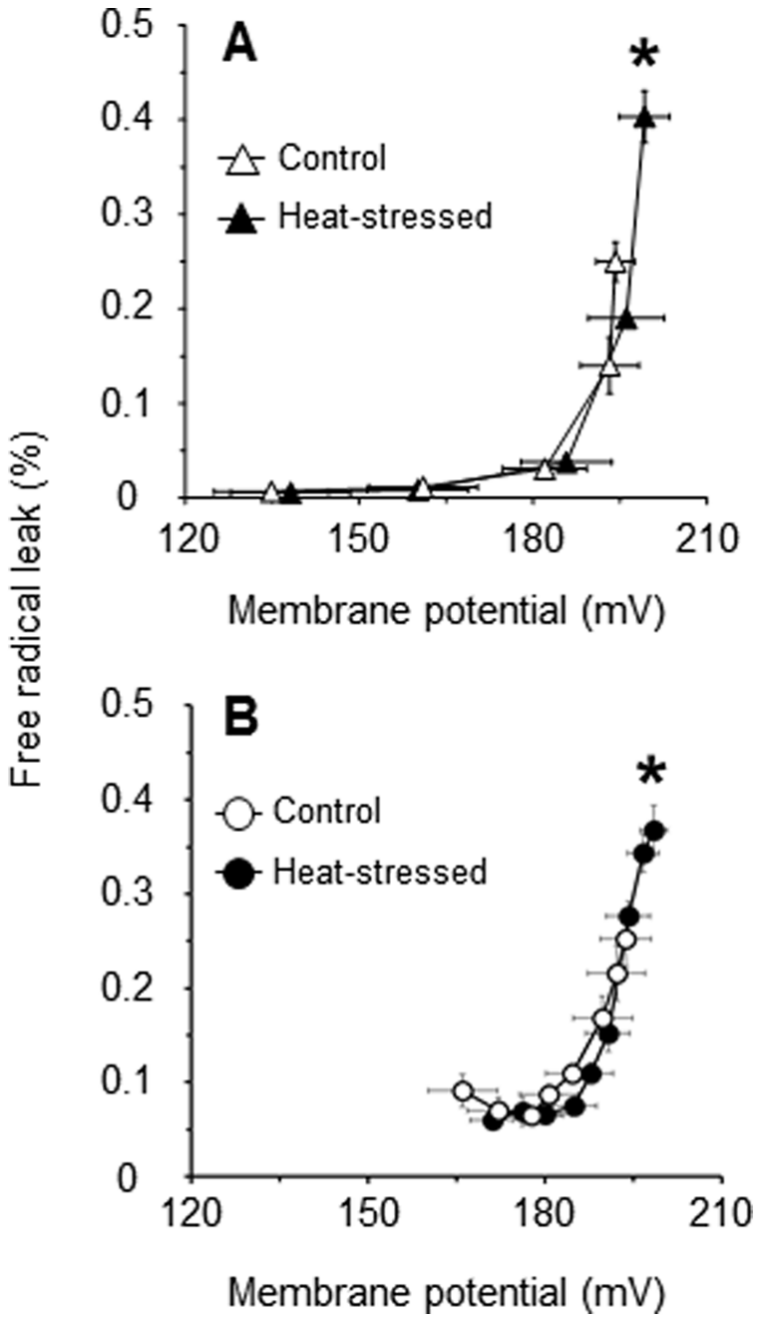
Relation of mitochondrial free radical leak (FRL) to ΔΨ for substrate oxidation (A) and basal proton leak (B) in skeletal muscle mitochondria of control (open symbols) and heat-stressed (closed symbols) groups. The FRL was calculated as the ratio of H_2_O_2_ production rate to twice the O_2_ consumption rate, and the result expressed as a percentage (for details see Methods). Values are means ± S.E. of data from four individual measurements in each group. *P<0.05 compared with the controls in the FRL.

The mitochondrial O_2_ consumption rate is another possible factor underlying the ROS production, with a higher O_2_ consumption rate giving rise to an increased ROS production. To test this point, mitochondrial FRL (H_2_O_2_ production rate/2× O_2_ consumption rate [30]) was calculated to more precisely evaluate the role of ΔΨ in the acute heat stress-induced overproduction of ROS. As shown in Fig. 3A, the percentage FRL for the substrate oxidation correlated exponentially with ΔΨ both in the control and heat-stressed groups. The FRL for basal proton leak also correlated exponentially with ΔΨ in both groups, even though there was a small difference in the FRL at any given ΔΨ (Fig. 3B). For both substrate oxidation and basal proton leak kinetics, the highest value of FRL was found in state 4 (furthest point to the right in the kinetic curve) (Fig. 3A, B). Moreover, the FRL in state 4 was significantly higher in the heat-stressed group than in control group for each kinetic response, which corresponds to the increased ΔΨ for the heat-stressed group compared with controls (Fig. 3A, B). Thus, it can be concluded that an increase of ΔΨ serves as a major determinant of the overproduction of ROS in the skeletal muscle mitochondria of heat-stressed birds.

### Mitochondrial ROS production and ΔΨ with complex I- and/or complex II-linked substrates

**Figure 4:**
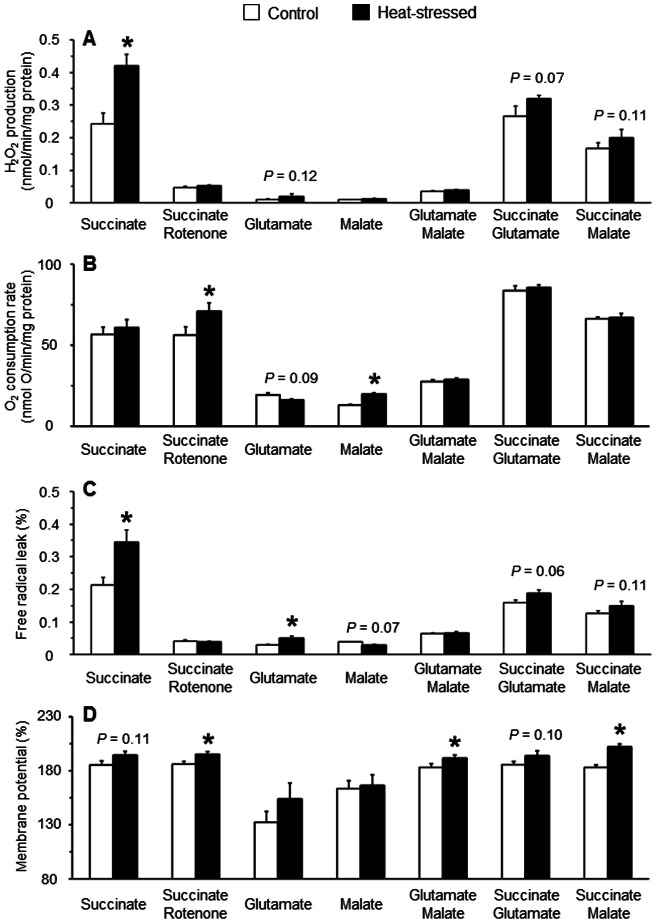
H_2_O_2_ production (A), O_2_ consumption rate (B), free radical leak (C) and ΔΨ (D) in muscle mitochondria energized with complex I- and/or complex II-linked substrates. Mitochondria (0.5 mg protein/ml) were incubated in assay medium B containing 0.1 μM nigericin and 1 μg/ml oligomycin, to which 10 mM glutamate and 2.5 mM malate as complex I-linked substrates and 4 mM succinate (in the presence or absence of 5 μM rotenone) as a complex II-linked substrate were used to initiate respiration. Mitochondrial H_2_O_2_ production, ΔΨ and oxygen consumption rate were measured as previously described (see Methods for details). Values are means ± SE of data from four individual measurements in each group. *P<0.05 compared with the controls.

In the above (Fig. 2D), we obtained direct evidence that the acute heat stressed-induced overproduction of mitochondrial ROS depends extensively on ΔΨ when mitochondria were energized with succinate as a complex II-linked substrate. To determine whether the overproduction of mitochondrial ROS due to the heat stress is substrate-dependent, we measured the ROS production when mitochondria were energized with complex I-linked substrate alone or in combination with succinate. In agreement with the results of Fig. 2D, mitochondrial H_2_O_2_ production with succinate alone was significantly increased by the heat exposure; however this increase did not occur in the presence of rotenone (Fig. 4A). Levels of H_2_O_2_ production in both control and heat-stressed groups in the presence of glutamate, malate or glutamate/malate were significantly lower than that when succinate alone was used (Fig. 4A). There were no differences between the groups in terms of H_2_O_2_ production with either malate or glutamate/malate, while the H_2_O_2_ production with glutamate tended to be higher (*P* = 0.12) in the heat-stressed group compared with the control group. Interestingly, H_2_O_2_ production with either glutamate or malate was markedly increased by the simultaneous addition of succinate both in the control and heat-stressed groups. Furthermore, the H_2_O_2_ production with either succinate/glutamate or succinate/malate tended to be higher in the heat-stressed group compared with the control group, though this was not statistically significant (*P* = 0.07 and 0.11, respectively).

The O_2_ consumption rate with succinate alone was not different between control and heat-stressed groups, but was significantly higher in the heat-stressed group when mitochondria were incubated in the presence of rotenone (Fig. 4B). O_2_ consumption rates in both control and heat-stressed groups in the presence of glutamate, malate or glutamate/malate were significantly lower than that when succinate alone was used. Moreover, the O_2_ consumption rate with glutamate tended to be decreased in the heat-stressed group compared with the control group (*P* = 0.09), while the O_2_ consumption rate with malate was significantly increased in the heat-stressed group. Furthermore, there was no difference between the groups in the O_2_ consumption rate with glutamate/malate. As was the case with mitochondrial H_2_O_2_ production, O_2_ consumption rates in the presence of glutamate or malate were significantly increased by the addition of succinate in both the control and heat-stressed groups. There was no difference in the O_2_ consumption rate with either succinate/glutamate or succinate/malate between the groups (Fig. 4B).

In agreement with the results of Fig. 3, the FRL for succinate alone was significantly higher in the heat-stressed group than in the control group, whereas little difference in the FRL for succinate in the presence of rotenone was observed between the groups (Fig. 4C). The FRL for glutamate was significantly higher in the heat-stressed group than in control group given that the O_2_ consumption rate with glutamate was increased in the heat-stressed group. In contrast, the FRL for malate was tended to be lower in the heat-stressed group than in the control group (*P* = 0.07) as the O_2_ consumption rate with malate was increased by the heat exposure. In addition, there were only small differences observed in the FRL for glutamate/malate between the groups. Finally, the FRL for succinate/glutamate and succinate/malate tended to be higher in the heat-stressed group (*P* = 0.06 and 0.11, respectively).

The ΔΨ for succinate-driven oxidation tended to be increased in the heat-stressed group compared with the control group (*P* = 0.11), and this increase was also observed in the presence of rotenone (Fig. 4D). These results suggest that in the presence of rotenone, H_2_O_2_ production with succinate was not increased by the heat exposure even though the ΔΨ was significantly increased. ΔΨ for glutamate was considerably lower than that for succinate in both the control and heat-stressed groups, and appeared to be higher for the heat-stressed group compared with the control group, though these differences were not statistically significant. Furthermore, there was no difference between the groups with respect to the ΔΨ for malate-driven oxidation. Although no difference in the H_2_O_2_ production with glutamate/malate was observed between the groups, the ΔΨ for the substrates was significantly increased in the heat-stress group compared with the controls. Thus, it is likely that ROS production due to complex I-linked substrates such as glutamate and glutamate/malate in heat-stressed birds is ΔΨ-independent. Further to this, the ΔΨ for succinate/glutamate showed a tendency to be increased in the heat-stressed group compared with the control group (*P* = 0.10), while the ΔΨ for succinate/malate was significantly increased in the heat-stressed group compared with the control group (Fig. 4D). These results suggest that the excess ROS production with either succinate/glutamate or succinate/malate in the heat-stressed group might be affected by the increase of ΔΨ, but not by an increased O_2_ consumption.

### Mitochondrial avUCP protein content and avUCP-mediated proton leak

**Figure 5:**
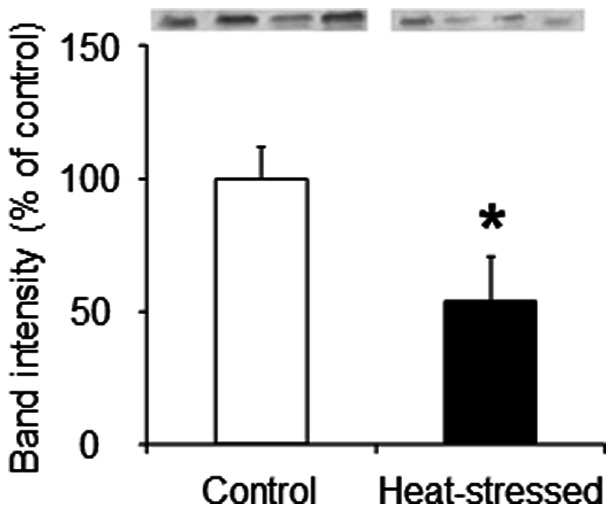
Mitochondrial avUCP protein levels in skeletal muscle. Immunodetection of mitochondrial avUCP protein content was estimated by Western blot analyses of mitochondrial protein (50 μg) using an anti-avUCP antibody. Values are means ± S.E. of four individual mitochondrial samples. *P<0.05 compared with control.

We evaluated the effect of a heat stress-induced decrease of avUCP content on proton leak and ΔΨ. In agreement with previous studies [18], [19], the avUCP protein content of muscle mitochondria was significantly decreased by the heat stress (Fig. 5).

**Figure 6:**
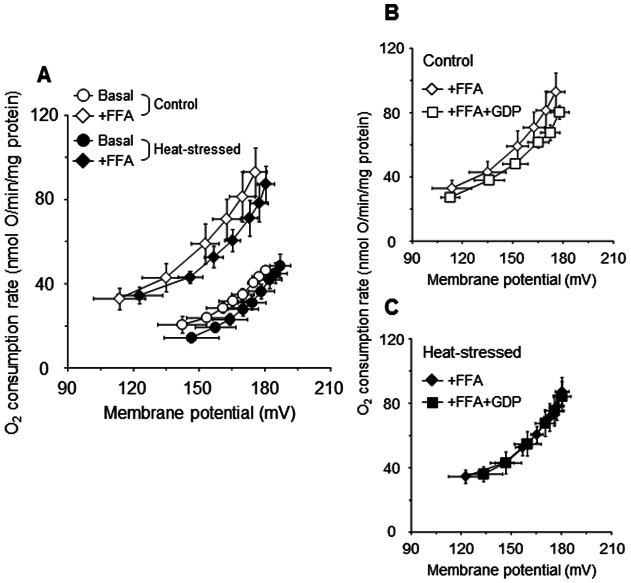
Effect of arachidonic acid and GDP on proton leak kinetics of skeletal muscle mitochondria isolated from control (open symbols) and heat-stressed (filled symbols) birds. Basal (circles) and arachidonic acid-induced (36 μM, diamonds) proton leak kinetics in both groups (A). Inhibitory effect of GDP (500 μM, squares) on the arachidonic acid-induced proton leak in control (B) and heat-stressed (C) groups. Mitochondria (0.35 mg protein/ml) were energized by 4 mM succinate in assay medium B containing 0.1 μM nigericin and 1 μg/ml oligomycin, and titrated with sequential additions of malonate (up to 3.2 mM). Value are means ± S.E. of four individual measurements in each group.

Figure 6A shows that the basal proton leak was slightly reduced in the heat-stressed group compared with the control group for any given ΔΨ, and that this leak was considerably increased in both groups (but less-so for heat-stressed birds) following the addition of arachidonic acid. This finding indicates that the uncoupling effect due to the arachidonic acid was reduced in the heat-stressed group compared with the control group. In addition, the arachidonic acid-induced proton leak was slightly but unquestionably inhibited by GDP in the control group (Fig. 6B, open squares), but not in the heat-stressed group (Fig. 6C, filled squares). In this way, the heat stress resulted in a reduced GDP-sensitive portion of the arachidonic acid-stimulated uncoupled respiration. It is thus conceivable that the decrease in avUCP protein content due to the heat stress might result in the reduction of avUCP-mediated proton leak in the skeletal muscle mitochondria of birds.

Furthermore, we confirmed the effect of a reduction of the proton leak on ΔΨ at the furthest right-hand point in the kinetics curves. The value of ΔΨ at the furthest point to the right of the kinetic curve of arachidonic acid-induced proton leak for heat-stressed birds (Fig. 6) was slightly but not statistically significantly increased (+4.8 mV) compared with the control value. From these results, it is conceivable that the reduction of proton leak due to a decrease in avUCP protein content results in an increase of ΔΨ, which could be associated with the overproduction of mitochondrial ROS in the skeletal muscle of birds exposed to acute heat stress.

## Discussion

The present study confirms that acute heat stress induces mitochondrial ROS production, resulting in skeletal muscle oxidative damage in birds exposed to such conditions. We thus focused our attention in this study on the mechanism underlying the overproduction of mitochondrial ROS in response to heat exposure.

This study clarified that the overproduction of mitochondrial ROS with succinate-driven oxidation in heat-stressed birds results from an increase of ΔΨ (Fig. 2D). Furthermore, this study revealed that an increase in ROS production and ΔΨ with succinate in the presence of NADH-linked substrates such as glutamate or malate was observed in the heat-stressed group (Fig. 4A, 4D). Recently, Muller et al. [33] reported that mitochondrial ROS production in the presence of succinate with NADH-linked substrates was completely abolished by the addition of FCCP, thereby depolarizing ΔΨ. It is therefore evident that the increased ΔΨ together with succinate oxidation may play a pivotal role in the overproduction of mitochondrial ROS due to heat stress. Regarding the mechanism of how these factors affect the overproduction of mitochondrial ROS in the skeletal muscle of heat-stressed birds, the fact that complex I of the electron transport chain can produce superoxide at relatively high rates during succinate oxidation [34], [35] should be kept in mind; this phenomenon is probably due to reverse electron flow. In addition, ROS production with succinate depends extensively on ΔΨ [14], [15]. It could thus be postulated that the overproduction of ROS with succinate in heat-stressed birds may be involved in the reverse electron flow. This possibility is supported by the fact that the ROS production with succinate in the presence of rotenone, which can prevent the reverse electron flow, was not increased by the heat exposure (Fig. 4A) even though ΔΨ was significantly increased (Fig. 4D). Therefore, it can be concluded that the reverse electron flow during succinate oxidation could also be a determinant of the overproduction of mitochondrial ROS in the skeletal muscles of heat-stressed birds.

One of the issues in the study of mitochondria is whether reverse electron flow-mediated ROS production actually occurs under substrate conditions that are physiologically plausible. In the present study, NADH- and FADH_2_-linked substrates were simultaneously added to initiate mitochondrial respiration, which might be physiologically more realistic compared to conditions where either NADH- or FADH_2_-linked substrates are added. It is thus conceivable that a reverse electron flow-mediated overproduction of mitochondrial ROS could occur in skeletal muscle mitochondria of heat-stressed birds.

It should be noted that mitochondrial ROS production with glutamate was increased by acute heat stress; this could be associated with the oxidative damage, even though the degree of ROS overproduction for glutamate was appreciably lower compared to that for succinate (Fig. 4A). Interestingly, the mitochondrial FRL for glutamate was increased by the heat exposure (Fig. 4C) without any change in the ΔΨ (Fig. 4D). From these results, it can be postulated that not only ΔΨ but also the respiratory rate are not involved in the overproduction of ROS with NADH-linked substrates in the heat-stressed group. This postulation is supported by the fact that no difference in ROS production with malate was observed between the groups even though the O_2_ consumption rate for malate was significantly increased in the heat-stressed group (Fig. 4A, B). Thus, another factor might be responsible for the overproduction of ROS with glutamate in the heat-stressed birds. One could postulate that α-ketoglutarate dehydrogenase, which has been recently reported as a site for ROS production in mitochondria [11], [36], might be associated with the ROS overproduction with glutamate in response to the heat stress. However, the possible involvement of this enzyme in the overproduction of mitochondrial ROS remains poorly understood both for physiological and pathological conditions. Further experiments are required to determine the possible mechanisms for the glutamate-supported overproduction of mitochondrial ROS due to heat stress.

No difference in ROS production with glutamate/malate was observed between the control and heat-stressed groups in this study (Fig. 4A). This observation is not in accordance with a previous finding that mitochondrial ROS production with glutamate/malate was significantly increased by acute heat stress in avian skeletal muscle [9]. Whether this discrepancy arises from the ages of birds employed or from the use of different assay systems to measure the mitochondrial ROS production is not clear. Regarding the age, the present study used 21-day-old birds exposed to acute heat stress, whereas 16-day-old birds were used in the previous study [9], [19]: The average body weight of birds when exposed to heat in this study was approximately 35% greater than that for the previous study.

A small amount of information is available regarding the function of avUCP for proton leak in avian muscle mitochondria. Rey et al. (2010) have found that the activity of avUCP in mitochondria is related to the relative abundance of the avUCP transcript [37]. The present study provides evidence for the first time that a decrease in avUCP protein content due to acute heat stress possibly reduces proton leak in the skeletal muscle mitochondria of birds (Fig. 6). It is therefore conceivable that avUCP might exert a similar function to that seen with mammalian UCP (especially UCP3). Considering the suggestion that activated proton leak reduces mitochondrial superoxide production by decreasing the proton motive force and the local oxygen concentration [21], [38], it might be postulated that the decrease in avUCP protein content may also be a determinant of the overproduction of mitochondrial ROS in heat-stressed birds. Goglia and Skulachev [39] hypothesized that UCPs participate in the export of lipid hydroperoxides (LOOH) outside the mitochondrial matrix. This hypothesis was validated by Lombardi et al. [40], [41], whereupon it was shown that LOOH induce UCP3-mediated uncoupling, thereby leading to the export of LOOH across the mitochondrial inner membrane. In a manner similar to that of Lombardi et al. [41], avUCP-mediated proton leak was measured in the present study by using arachidonic acid (and GDP) in the absence of rotenone; LOOH can be formed from arachidonic acid and endogenously generated ROS via reverse electron flow. As a result, the GDP-sensitive portion of arachidonic acid-induced uncoupling was substantially reduced in response to the heat stress (Fig. 6C), which may result from the decrease in avUCP protein under these conditions (Fig. 5). Given that superoxide activates UCPs [29], it cannot be ruled out that endogenously-generated superoxide in the absence of rotenone could also induce the uncoupling effects in avian muscle mitochondria.

Moreover, the fact that not only UCP but also adenine nucleotide translocator (ANT) can mediate uncoupling by free fatty acids [42]–[44] allows us to postulate that changes in ANT expression might affect the arachidonic acid-induced proton leak. Carboxyatractyloside, which is a strong inhibitor of ANT, was not added to the assay medium in the present study, meaning that a possible involvement of ANT in the arachidonic acid-induced proton leak in skeletal muscle mitochondria of birds cannot be ruled out. However, we have previously found that the avANT transcript was not altered by heat treatments [16], [18], [19], indicating that avANT probably does not affect the reduction of inducible proton leak in heat-stressed birds. It can therefore be concluded that acute heat stress decreases the mitochondrial avUCP protein content, resulting in a reduction of the avUCP-mediated proton leak in the skeletal muscle of heat-stressed birds.

Despite the above finding concerning avUCP content, we did not evaluate the effect of arachidonic acid-induced proton leak on mitochondrial ROS production in heat-stressed birds in the present study. However, a possible explanation for the ROS overproduction due to the reduction of avUCP-mediated proton leak can be provided. From the results showing that the value of ΔΨ at the furthest point to the right in the kinetic curve was slightly increased due to heat exposure (Fig. 6), one may postulate that the slight increase in ΔΨ might increase ROS production in heat-stressed birds. As shown in Fig. 2D, mitochondrial ROS production was correlated exponentially with the elevation of ΔΨ: a 2.7% (+5.3 mV) increase in ΔΨ in state 4 for the heat-stressed groups led to a 47% (+0.12 nmol H_2_O_2_/min/mg protein) statistically significant increase in ROS production, suggesting that even if the increase in ΔΨ is quite small, the ROS production is significantly increased in heat-stressed birds. Thus, it is conceivable that the slight increase in ΔΨ due to the decrease in avUCP-mediated proton leak could induce mitochondrial ROS production in heat-stressed birds.

Here, we have shown that *i*) the overproduction of mitochondrial ROS with succinate-driven oxidation may depend on an increase of ΔΨ in the skeletal muscle mitochondria of birds exposed to acute heat stress. We also found that *ii*) mitochondrial ROS production was increased when mitochondria were energized in the presence of succinate with either glutamate or malate, probably due to an increase in ΔΨ, indicating that ROS overproduction may occur under physiologically relevant conditions. Moreover, *iii*) a decrease in avUCP protein content due to the heat exposure possibly reduces the avUCP-mediated proton leak, which might be associated with the overproduction of mitochondrial ROS, possibly via the increase of ΔΨ.
