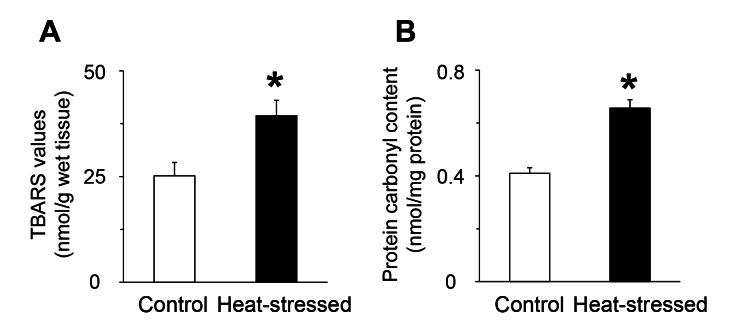# Correction: Crucial Role of Membrane Potential in Heat Stress-Induced Overproduction of Reactive Oxygen Species in Avian Skeletal Muscle Mitochondria

**DOI:** 10.1371/annotation/6f622a41-940c-4c12-a82e-ea023cd61e81

**Published:** 2013-05-22

**Authors:** Motoi Kikusato, Masaaki Toyomizu

The incorrect version of Figure 1 appeared in the article. The correct version is available here: 

**Figure:**